# Efficient Degradation of Aflatoxin B_1_ and Zearalenone by Laccase-like Multicopper Oxidase from *Streptomyces thermocarboxydus* in the Presence of Mediators

**DOI:** 10.3390/toxins13110754

**Published:** 2021-10-24

**Authors:** Xing Qin, Yanzhe Xin, Jiahuan Zou, Xiaoyun Su, Xiaolu Wang, Yaru Wang, Jie Zhang, Tao Tu, Bin Yao, Huiying Luo, Huoqing Huang

**Affiliations:** State Key Laboratory of Animal Nutrition, Institute of Animal Sciences, Chinese Academy of Agricultural Sciences, Beijing 100193, China; qinxing@caas.cn (X.Q.); xyanzhe@163.com (Y.X.); zjh0512@126.com (J.Z.); suxiaoyun@caas.cn (X.S.); wangxiaolu@caas.cn (X.W.); wangyaru@caas.cn (Y.W.); zhangjie09@caas.cn (J.Z.); tutao@caas.cn (T.T.); yaobin@caas.cn (B.Y.)

**Keywords:** multicopper oxidase, mycotoxin, aflatoxin, zearalenone, degradation, mediator

## Abstract

Multicopper oxidases (MCOs) are a diverse group of enzymes that could catalyze the oxidation of different xenobiotic compounds, with simultaneous reduction in oxygen to water. Aside from laccase, one member of the MCO superfamily has shown great potential in the biodegradation of mycotoxins; however, the mycotoxin degradation ability of other MCOs is uncertain. In this study, a novel MCO-encoding gene, *StMCO*, from *Streptomyces thermocarboxydus,* was identified, cloned, and heterologously expressed in *Escherichia coli*. The purified recombinant *St*MCO exhibited the characteristic blue color and bivalent copper ion-dependent enzyme activity. It was capable of oxidizing the model substrate ABTS, phenolic compound DMP, and azo dye RB5. Notably, *St*MCO could directly degrade aflatoxin B_1_ (AFB_1_) and zearalenone (ZEN) in the absence of mediators. Meanwhile, the presence of various lignin unit-derived natural mediators or ABTS could significantly accelerate the degradation of AFB_1_ and ZEN by *St*MCO. Furthermore, the biological toxicities of their corresponding degradation products, AFQ_1_ and 13-OH-ZEN-quinone, were remarkably decreased. Our findings suggested that efficient degradation of mycotoxins with mediators might be a common feature of the MCOs superfamily. In summary, the unique properties of MCOs make them good candidates for degrading multiple major mycotoxins in contaminated feed and food.

## 1. Introduction

Mycotoxins are toxic fungal secondary metabolites that are widely distributed in contaminated feed and food, bringing about many adverse health effects on livestock and humans, as well as huge economic losses in animal husbandry and the food industry [1]. As of now, there are hundreds of types of mycotoxins that have been identified, but the most frequently observed mycotoxins in contaminated feed and food are aflatoxin B_1_ (AFB_1_), zearalenone (ZEN), deoxynivalenol, fumonisin B_1_, and ochratoxin A [2]. AFB_1_ is mainly produced by *Aspergillus flavus* and *A. parasitica*, displaying carcinogenic, teratogenic, and immunosuppressive toxicity [3], and has been recognized as a group I carcinogen by the International Agency for Research on Cancer [4]. ZEN is primarily produced by *Fusarium graminearum*, *F. culmorum*, *F. cerealis*, *F. equiseti*, and *F. verticillioides*, exerting reproductive toxicity, hepatotoxicity, immunotoxicity, and genotoxicity [5,6]. Moreover, according to the Food and Agriculture Organization of the United Nations report, about 25% of global food crops are contaminated with these mycotoxins, resulting in an economic loss of billions of dollars each year [7]. Therefore, efficient mycotoxin detoxification strategies are in great demand.

In comparison with traditional physical and chemical detoxification methods, the biological detoxification of mycotoxins using microorganisms and enzymes is one of the most promising methods because of its high efficiency, irreversibility, and environmental friendliness [8]. During the past three decades, a variety of pre- and post-harvest biological control strategies have been developed to reduce mycotoxin contamination in feed and food [9,10,11,12]. On the one hand, bacteria, such as *Bacillus* and *Pseudomonas,* and fungi belonging the genus *Trichoderma* are used as the main biocontrol agents to limit the growth of mycotoxin-producing molds at the pre-harvest stage [9]. On the other hand, different microorganisms, including bacteria, yeast, and fungi, as well as their enzymes, are adopted to transform mycotoxins into less toxic or nontoxic metabolites during the post-harvest period [12].

In recent years, the degradation of mycotoxins with ligninolytic microorganisms and their corresponding ligninolytic enzymes has received more and more attention from researchers [13,14,15,16,17,18]. Interestingly, the broad substrate specificity of ligninolytic enzymes enables them to degrade different structural types of mycotoxins, including AFB_1_, ZEN, deoxynivalenol, fumonisin B_1_, and ochratoxin A [16,17]. Meanwhile, ligninolytic enzymes, such as laccase and dye-decolorizing peroxidase, can significantly accelerate the degradation of mycotoxins in the presence of mediators [19,20]. These catalytic properties of ligninolytic enzymes make them promising candidates for mycotoxin degradation. Significantly, laccase belongs to a member of the multicopper oxidase superfamily that contains laccase, laccase-like multicopper oxidase, ferroxidase, and so on [21], and it is not yet clear whether other MCOs could be able to degrade multiple major mycotoxins. In addition, there is a lack of systematic assessments of lignocellulose-derived compounds as the natural mediators of MCOs for mycotoxin degradation.

*Streptomyces* species are well-known bacteria capable of lignin degradation, and their ligninolytic enzyme system comprises multicopper oxidase, dye-decolorizing peroxidase, and lignin peroxidase, based on the genome-wide annotation analysis [20,22]. In this study, a novel laccase-like multicopper oxidase, *St*MCO, from *Streptomyces thermocarboxydus* 41291, was heterogeneously expressed, purified, and biochemically characterized. Moreover, the AFB_1_ and ZEN degradation properties of purified recombinant *St*MCO, in the presence of different structural lignin model compounds or ABTS, were analyzed and evaluated. Furthermore, their degradation products were identified by UPLC-MS/MS.

## 2. Results and Discussion

### 2.1. Gene Cloning and Sequence Analysis of StMCO from S. thermocarboxydus

It had been reported that the ligninolytic enzyme system of *S. thermocarboxydus* 41291 consisted of multicopper oxidase and dye-decolorizing peroxidase [20]. In this study, one novel multicopper oxidase-encoding gene, *StMCO*, was cloned from the genomic DNA of *S. thermocarboxydus* 41291. It was composed of a 990 bp open reading frame encoding 329 amino acid residues with a calculated molecular weight of approximately 36 kDa. The deduced amino acid sequence contained a putative twin-arginine signal peptide of 31 amino acid residues for secretory expression. Based on the BLAST search in NCBI, *St*MCO only contained two cupredoxin domains, while most multicopper oxidases consisted of three domains [23,24]. Additionally, each cupredoxin domain of *St*MCO encompassed one copper binding site. The multiple sequence alignment further revealed that there was one T2/T3 trinuclear copper binding site (two conserved HxH motifs) and one T1 copper binding site (conserved HxxHxH and HCHxxxH motifs) in the first and second cupredoxin domain, respectively (Figure S1). Moreover, according to the number and location of the T1 copper binding sites, the two-domain multicopper oxidase (2dMCO) superfamily was subdivided into the following three subfamilies: A, B, and C [24]. Type A 2dMCO contained two T1 copper binding sites. In contrast, type B and C 2dMCO included one T1 copper binding site in the second and first domain, respectively. Taken together, *St*MCO belonged to type B 2dMCO.

### 2.2. Expression and Purification of StMCO

**Figure 1:**
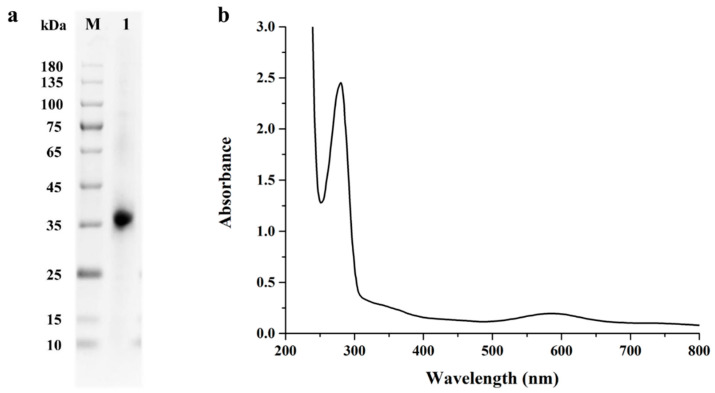
The SDS-PAGE (**a**) and UV–visible spectroscopy (**b**) analysis of purified recombinant *St*MCO from *S. thermocarboxydus*. Lane M, the protein molecular mass marker; lane 1, the purified recombinant *St*MCO.

Given that *Escherichia coli* was the most popular approach for producing recombinant proteins, the recombinant plasmid pCold I-*St*MCO was transformed into *E. coli* Transetta (DE3) for heterologous expression. After isopropyl-β-D-thiogalactoside (IPTG) induction, there was obvious multicopper oxidase activity in the supernatant of the cell lysates, indicating that *St*MCO was successfully heterologously expressed in *E. coli* Transetta (DE3). After purification via nickel-immobilized metal ion affinity chromatography, a strong blue color was observed in the eluted fraction. Meanwhile, the SDS-PAGE analysis of the eluted fraction corresponding to *St*MCO exhibited a signal protein band at ~36 kDa (Figure 1a). Significantly, *St*MCO was found to generate a multimer with a molecular mass >100 kDa in native PAGE (Figure S2), which was consistent with the structural feature of active 2dMCO in multimeric forms (homotrimer or homohexamer) [25,26].

### 2.3. Biochemical Characterization of Purified Recombinant StMCO

Multicopper oxidase was reported to include at least two highly conserved copper centers, which are as follows: one T1 copper center for substrate oxidation and one T2/T3 trinuclear copper center for oxygen reduction [27]. Notably, the T1 copper center exhibited an absorption peak around 600 nm, giving rise to the characteristic blue color [28]. As shown in Figure 1b, the UV–visible spectrum of purified recombinant *St*MCO displayed a maximum absorption peak at ~600 nm, indicating that it was a typical blue multicopper oxidase.

**Figure 2:**
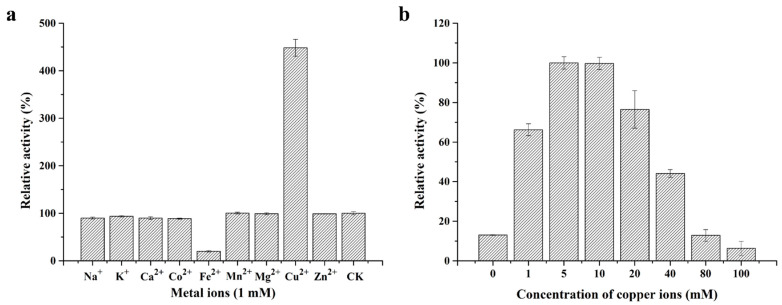
The effect of metal ion (**a**) and copper ion concentration (**b**) on the activity of purified recombinant *St*MCO.

Considering that multicopper oxidases used metal ions as cofactors to oxidize different substrates [29], the effect of metal ions, at a concentration of 1 mM, on the activity of purified recombinant *St*MCO was evaluated. As shown in Figure 2a, most metal ions, such as Na^+^, K^+^, Ca^2+^, Co^2+^, Mn^2+^, Mg^2+^, and Zn^2+^, had no effect on multicopper oxidase activity. It was worth noting that Cu^2+^ could remarkably increase enzymatic activity by 348%, while Fe^2+^ displayed 80% inhibition of enzymatic activity. Moreover, the enzymatic activity of *St*MCO was further enhanced with increasing Cu^2+^ concentrations, and reached its maximum in the range of 5 to 10 mM Cu^2+^ (Figure 2b). When the concentration of copper ions was higher than 10 mM, the multicopper oxidase activity began to decrease, suggesting that the optimal Cu^2+^ concentration was 5−10 mM for maximal activity. These results were in agreement with those obtained from previous studies that found that increased copper concentrations led to higher enzymatic activity because Cu^2+^ was the essential cofactor for multicopper oxidases [29,30,31].

**Figure 3:**
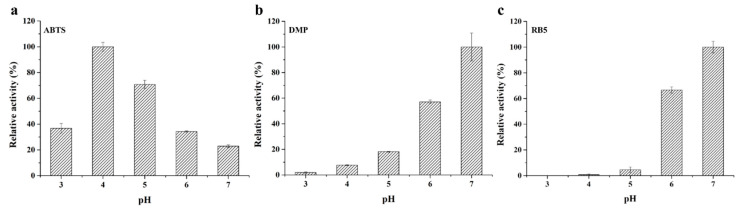
The optimum pH of purified recombinant *St*MCO for the oxidation of the following different substrates: ABTS (**a**), DMP (**b**), and RB5 (**c**).

Multicopper oxidases, particularly laccase, were able to catalyze reactions involving a broad range of substrates, such as the model substrate 2,2′-azino-bis(3-ethylbenzothiazoline-6-sulfonic acid) (ABTS), lignin-related aromatic compounds, metal ions, and so on [32]. The purified recombinant *St*MCO was capable of oxidizing various substrates, including the model substrate ABTS, phenolic compound 2,6-dimethylphenol (DMP), and azo dye reactive black 5 (RB5), but it could not oxidize the phenolic compound guaiacol (GUA), non-phenolic compound veratryl alcohol (VA), and anthraquinone dye reactive blue 19 (RB19), which was similar to the substrate specificity of most other reported bacterial laccases [33,34]. However, the accurate classification of multicopper oxidase, assigned as laccase, still remained unclear [33], thus *St*MCO was termed a laccase-like multicopper oxidase. In addition, the optimum pH for the oxidation of different substrates by *St*MCO was 4.0 for ABTS, 7.0 for DMP, and 7.0 for RB5, respectively (Figure 3), exhibiting a substrate-dependent shift of optimum pH. The specific activity of purified recombinant *St*MCO towards ABTS, DMP, and RB5 at optimum pH was 0.259±0.009, 0.207±0.023, and 0.051±0.002 U/mg, respectively. Surprisingly, the specific activity of *St*MCO against DMP was one order of magnitude lower than that of ABTS, which might be attributed to the different bisubstrate reaction mechanism. It was reported that the bisubstrate models of ABTS and DMP oxidation by multicopper oxidases were ping-pong and Theorell–Chance, respectively [35].

### 2.4. Enzymatic Degradation of AFB_1_ and ZEN by StMCO

Recently, several laccases have been reported to be able to degrade multiple major mycotoxins, such as AFB_1_ and ZEN, in the presence of various mediators [19,36,37]. However, it was not clear whether mycotoxin degradation is the common feature of the multicopper oxidase superfamily. Besides, lignin-derived compounds as the natural mediators of MCOs for mycotoxin degradation lacked systematic evaluation. Herein, the degradation capacity of AFB_1_ and ZEN by the laccase-like multicopper oxidase *St*MCO, in the absence and presence of various structural lignin unit-derived mediators, was further evaluated.

**Figure 4:**
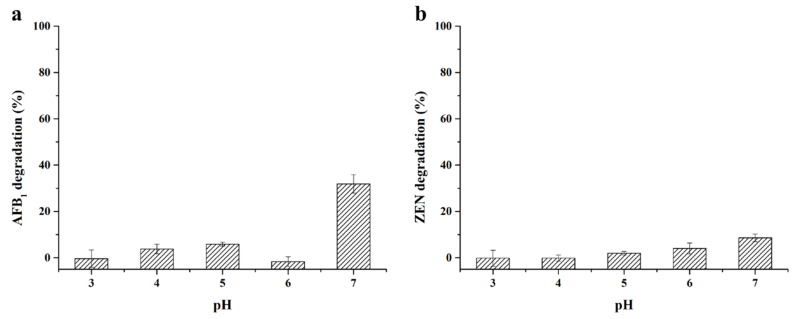
The optimum pH of purified recombinant *St*MCO for direct degradation of AFB_1_ (**a**) and ZEN (**b**) in 50 mM acetate buffer supplemented with 5 mM CuSO_4_ for 24 h at 30 °C.

As reported, Lac2 from *Pleurotus pulmonarius* [36], Ery4 from *P. eryngii* [37], and *Bs*CotA from *Bacillus subtilis* [19] were not able to directly degrade mycotoxins. However, as shown in Figure 4, *St*MCO could directly degrade AFB_1_ and ZEN in the absence of mediators, with pH 7 being the optimum pH. The degradation percentage of AFB_1_ and ZEN after the 24 h reaction was 31.87 ± 3.99% and 8.58 ± 1.63%, respectively, suggesting that enzyme–substrate interactions might exist between *St*MCO and mycotoxins.

**Figure 5:**
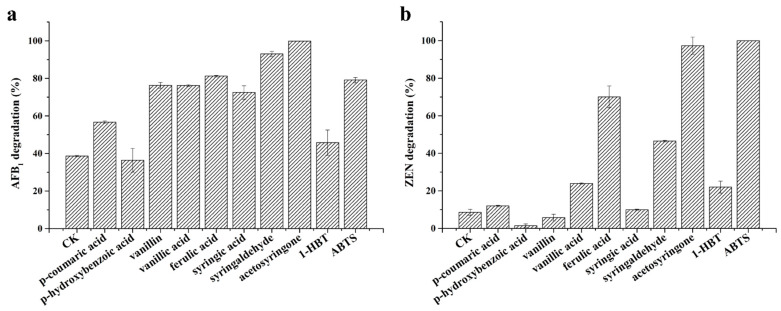
The effect of various mediators on the degradation of AFB_1_ (**a**) and ZEN (**b**) by 0.2 U/mL *St*MCO in 50 mM acetate buffer (pH 7.0) containing 1 mg/L AFB_1_ or ZEN, 5 mM CuSO_4_, and 1 mM mediator for 24 h at 30 °C.

Moreover, different lignin unit-derived natural mediators, including H-type monomers (*p*-coumaric acid and *p*-hydroxybenzoic acid), G-type monomers (vanillin, vanillic acid, and ferulic acid), S-type monomers (syringic acid, syringaldehyde, and acetosyringone), 1-hydroxybenzotriazole (1-HBT), and ABTS, were chosen to explore the effect on the degradation of AFB_1_ and ZEN by *St*MCO. As shown in Figure 5, most mediators were found to significantly increase the degradation percentage of AFB_1_ and ZEN. As for AFB_1_, acetosyringone was the best mediator, with 99.85% degradation, followed by syringaldehyde (93.03%), ferulic acid (81.19%), ABTS (79.11%), vanillin (76.26%), vanillic acid (76.22%), syringic acid (72.48%), and *p*-coumaric acid (56.66%), while *p*-hydroxybenzoic acid and 1-HBT were ineffective (Figure 5a). With regards to ZEN, ABTS was the best performing mediator, with a degradation percentage of 100%, followed by 97.35% for acetosyringone, 70.05% for ferulic acid, 46.53% for syringaldehyde, 23.98% for vanillic acid, and 21.96% for 1-HBT, but no improvement in the degradation of ZEN was observed for *p*-coumaric acid, *p*-hydroxybenzoic acid, vanillin, and syringic acid (Figure 5b). These results indicated that lignin unit-derived natural mediators might be alternative mediators for mycotoxin degradation by *St*MCO, in terms of the economic cost and environmental friendliness. Moreover, the great improvement in AFB_1_ and ZEN degradation in the presence of acetosyringone and ABTS might be attributed to the generation of high potential radicals, aryloxy radicals, and ABTS^++^, respectively [36]. Generally speaking, these results proved that *St*MCO might be a promising candidate for the efficient and simultaneous degradation of multiple major mycotoxins, with the use of a single or multiple mediators.

**Figure 6:**
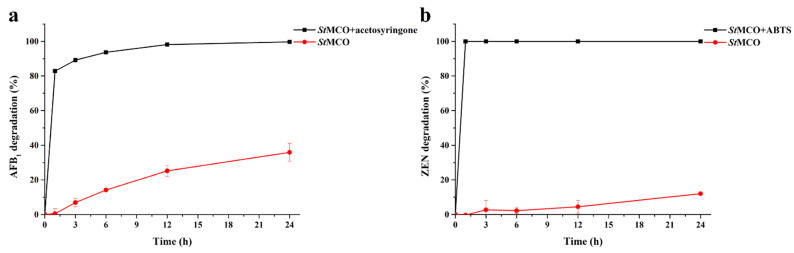
The time course analysis of AFB_1_ (**a**) and ZEN (**b**) degradation by 0.2 U/mL *St*MCO in 50 mM acetate buffer (pH 7.0) containing 1 mg/L AFB_1_ or ZEN, 5 mM CuSO_4_, and 1 mM acetosyringone or ABTS at 30 °C.

Furthermore, the time courses of AFB_1_ and ZEN degradation by *St*MCO, in the presence of their most efficient mediators, acetosyringone and ABTS, were determined. As shown in Figure 6, there was no significant change in the degradation of AFB_1_ and ZEN by *St*MCO in the absence of mediators after a 1 h reaction. In contrast, it was notable that AFB_1_ and ZEN were rapidly removed by *St*MCO in the presence of acetosyringone and ABTS, respectively.

### 2.5. Identification of AFB_1_ and ZEN Degradation Products

Considering that the biological detoxification of mycotoxins was defined as the degradation or enzymatic transformation of mycotoxins into less toxic or nontoxic compounds [38], the degradation products of AFB_1_ and ZEN by *St*MCO, in the presence of the most efficient mediator, were identified by UPLC-MS/MS, and their biological toxicities were further elucidated.

**Figure 7:**
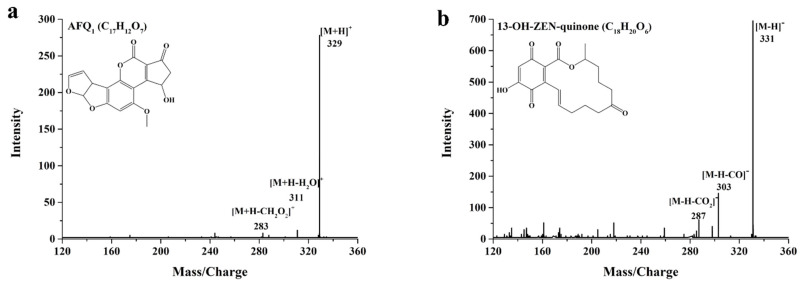
The UPLC-MS/MS analysis of AFB_1_ and ZEN degradation products, including AFQ_1_ (**a**) and 13-OH-ZEN-quinone (**b**) by 0.2 U/mL *St*MCO in 50 mM acetate buffer (pH 7.0) containing 1 mg/L AFB_1_ or ZEN, 5 mM CuSO_4_, and 1 mM acetosyringone or ABTS at 30 °C.

AFQ_1_ was the main degradation product of AFB_1_, corresponding to the parent ion at *m*/*z* 329 [M+H]^+^, and daughter ions at *m*/*z* 311 [M+H-H_2_O]^+^ and *m*/*z* 283 [M+H-CH_2_O_2_]^+^ (Figure 7a), though the AFQ_1_ content decreased slightly over time (Figure S3a). The identified degradation product AFQ_1_ was in accordance with the product of AFB_1_ degradation catalyzed by laccases [14,18], indicating that the ability to degrade mycotoxins might be the common feature of the multicopper oxidase superfamily. Notably, the toxic effects of AFQ_1_ on the chicken embryo were reported to be about 18 times less than AFB_1_ [39]. Moreover, AFQ_1_ had a lack of cytotoxicity in the human liver cells L-02 [18]. In addition, 13-OH-ZEN-quinone was found to be the main intermediate degradation product of ZEN, exhibiting the parent ion at *m*/*z* 311 [M-H]^−^, and daughter ions at *m*/*z* 303 [M-H-CO]^−^ and *m*/*z* 287 [M-H-CO_2_]^−^ (Figure 7b). With the prolongation of the reaction time, 13-OH-ZEN-quinone was further metabolized (Figure S3b). As reported for the relationship between the chemical structure and biological toxicity, hydroxylation of the aromatic moiety in ZEN exhibited a remarkable decrease in estrogenic activity when compared with ZEN [40]. Therefore, the biological toxicity of 13-OH-ZEN-quinone might be less toxic than ZEN.

## 3. Conclusions

In this study, a novel laccase-like multicopper oxidase, *StMCO*, from *Streptomyces thermocarboxydus,* was identified, cloned, and heterologously expressed in *E. coli*. *St*MCO was a typical blue multicopper oxidase and showed copper-dependent enzyme activity. Most importantly, *St*MCO could not only directly degrade multiple major mycotoxins, including AFB_1_ and ZEN, but also accelerate the degradation of AFB_1_ and ZEN in the presence of various lignin unit-derived natural mediators and ABTS. Moreover, biological toxicities of their corresponding degradation products, AFQ_1_ and 13-OH-ZEN-quinone, were significantly removed. These findings indicated that MCOs could serve as a promising biological tool for degrading multiple major mycotoxins in contaminated feed and food.

## 4. Material and Methods

### 4.1. Substrates and Chemicals

Substrates ABTS, DMP, GUA, VA, RB5, RB19, AFB_1_, ZEN, and various structural mediators including *p*-coumaric acid, *p*-hydroxybenzoic acid, vanillin, vanillic acid, ferulic acid, syringic acid, syringaldehyde, acetosyringone, and 1-HBT were purchased from Sigma-Aldrich (St. Louis, USA). All other chemicals were of analytical grade and purchased from Sinopharm Chemical Reagent (Beijing, China).

### 4.2. Strains and Plasmid

*S. thermocarboxydus* 41291 was obtained from the Agricultural Culture Collection of China (Beijing, China). The *E. coli* strains Trans1-T1 and Transetta (DE3) were purchased from TransGen (Beijing, China). The expression vector pCold I was purchased from Takara (Beijing, China).

### 4.3. Cloning, Expression and Purification of StMCO

The *St*MCO-encoding gene devoid of its signal sequence was amplified from the mycelia of *S. thermocarboxydus* 41291 with gene-specific primers (*St*MCO-*Nde* I-F: 5′ ATCATCATATCGAAGGTAGG*CATATG*TCCACCACGGCGAGAACCGCG 3′; *St*MCO-*Xba* I-R: 5′ TTTTAAGCAGAGATTACCTA*TCTAGA*GTGCGCGTGCCCGG ACTTCTC 3′). The PCR product was then ligated into the expression vector pCold I predigested with *Nde* I and *Xba* I to generate the recombinant plasmid pCold I-*St*MCO, which was transformed into *E. coli* Trans1-T1 for cloning and sequencing. After confirmation by sequencing, the recombinant plasmid pCold I-*St*MCO was extracted and subsequently transformed into Transetta (DE3) expression strain.

The *E. coli* Transetta (DE3) transformant containing pCold I-*St*MCO was picked and grown in LB broth supplemented with 100 μg/mL ampicillin and 20 μg/mL chloramphenicol at 37 °C to OD_600_ of 0.8−1.0, followed by adding IPTG and CuSO_4_ at a final concentration of 0.5 and 1 mM, respectively. After induction at 16 °C for 12 h, the cells were harvested by centrifugation, resuspended in binding buffer (20 mM sodium phosphate, 500 mM NaCl, pH 7.4), and lysed by sonication. The sonicated supernatant containing recombinant *St*MCO was then purified by nickel-immobilized metal ion affinity chromatography. The purity of recombinant *St*MCO was verified by 12% SDS-PAGE.

### 4.4. Biochemical Characterization of StMCO

The multicopper oxidase activity was determined by monitoring the oxidation of ABTS (ε_420_ = 36,000 M^−1^·cm^−1^) at 420 nm in 50 mM acetate buffer containing 1 mM ABTS, 5 mM CuSO_4_, and the appropriate diluted enzyme solution. One unit (U) of enzyme activity was defined as the amount of enzyme that oxidized 1 μmol of ABTS per min at 25 °C.

The UV–visible spectrum of purified recombinant *St*MCO was measured in phosphate buffer (20 mM, pH 7.4) in the range from 230 to 800 nm. The effect of metal ions such as Na^+^, K^+^, Ca^2+^, Co^2+^, Fe^2+^, Mn^2+^, Mg^2+^, Cu^2+^, and Zn^2+^ at a concentration of 1 mM on the activity of purified recombinant *St*MCO was evaluated in 50 mM acetate buffer (pH 4.0). The effect of copper ion concentration ranging from 1 to 100 mM on the activity of purified recombinant *St*MCO was determined in 50 mM acetate buffer (pH 4.0). The substrate specificity of purified recombinant *St*MCO was studied for the oxidation of six different substrates ABTS, DMP (ε_470_ = 12,100 M^−1^·cm^−1^), GUA (ε_465_ = 49,600 M^−1^·cm^−1^), VA (ε_310_ = 9,300 M^−1^·cm^−1^), RB5 (ε_596_ = 30,000 M^−1^·cm^−1^), and RB19 (ε_595_ = 10,000 M^−1^·cm^−1^) in 50 mM acetate buffer with pH ranging from 3.0 to 7.0.

### 4.5. Enzymatic Degradation of AFB_1_ and ZEN by StMCO

The evaluation of *St*MCO for AFB_1_ and ZEN degradation ability was carried out in 50 mM acetate buffer with pH ranging from 3.0 to 7.0 containing 1 mg/L AFB_1_ or ZEN, 5 mM CuSO_4_, and 0.2 U/mL *St*MCO for 24 h at 30 °C.

The effect of mediators, including *p*-coumaric acid, *p*-hydroxybenzoic acid, vanillin, vanillic acid, ferulic acid, syringic acid, syringaldehyde, AS, 1-HBT, and ABTS, on AFB_1_ and ZEN degradation by *St*MCO was determined in 50 mM acetate buffer (pH 7.0) containing 1 mg/L AFB_1_ or ZEN, 5 mM CuSO_4_, 1 mM mediator, and 0.2 U/mL *St*MCO for 24 h at 30 °C.

The time course of AFB_1_ and ZEN degradation by *St*MCO in the presence of the most efficient mediator was determined in 50 mM acetate buffer (pH 7.0) containing 1 mg/L AFB_1_ or ZEN, 5 mM CuSO_4_, 1 mM AS or ABTS, and 0.2 U/mL *St*MCO for 1, 3, 6, 9, 12, and 24 h at 30 °C.

The degradation of AFB_1_ and ZEN was analyzed by high-performance liquid chromatography coupled to RF-20A fluorescence detector using previous analytical methods [16]. In addition, their degradation products were identified by UPLC-MS/MS methods described in our previous studies [16,20].

## Data Availability

The data presented in this study are available on request from the corresponding author.

## Supplementary Materials

The following are available online at https://www.mdpi.com/article/10.3390/toxins13110754/s1, Figure S1. The amino acid sequence alignment of *St*MCO with a two domain multicopper oxidase SLAC from *Streptomyces coelicolor*. Blue boxes indicate the copper binding motifs. Figure S2. The native PAGE analysis of *St*MCO from *S. thermocarboxydus*. Lane M, the protein molecular mass marker; lane 1, the purified recombinant *St*MCO. Figure S3. The time-course analysis of AFB_1_ and ZEN degradation products by UPLC-MS/MS, including AFQ_1_ (a) and 13-OH-ZEN-quinone (b).
