# Supplementary material for: Efficient Degradation of Aflatoxin B1 and Zearalenone by Laccase-like Multicopper Oxidase from Streptomyces thermocarboxydus in the Presence of Mediators
Source: Toxins (Basel). 2021 Oct 24;13(11):754. doi: 10.3390/toxins13110754 (PMC8621583; doi:10.3390/toxins13110754)
Supplement: Supplementary file 1 [file toxins-13-00754-s001.zip › toxins-1398675-supplementary.pdf]

Efficient degradation of aflatoxin B1 and zearalenone by laccase-like multicopper oxidase from *Streptomyces thermocarboxydus* in the presence of mediators

Xing Qin, Yanzhe Xin, Jiahuan Zou, Xiaoyun Su, Xiaolu Wang, Yaru Wang, Jie Zhang, Tao Tu, Bin Yao, Huiying Luo and Huoqing Huang

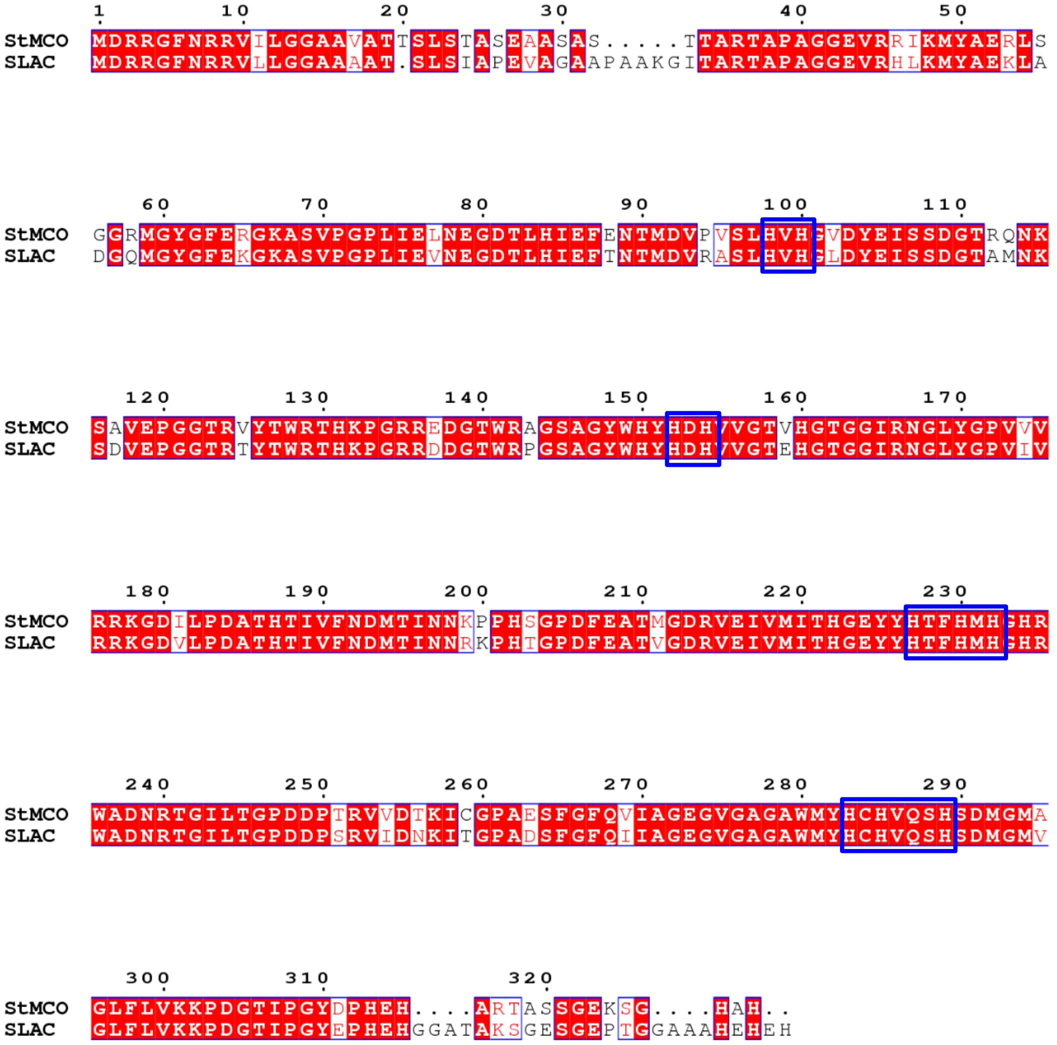

Figure S1. The amino acid sequence alignment of *St*MCO with a two domain multicopper oxidase SLAC from *Streptomyces coelicolor*. Blue boxes indicate the copper binding motifs.

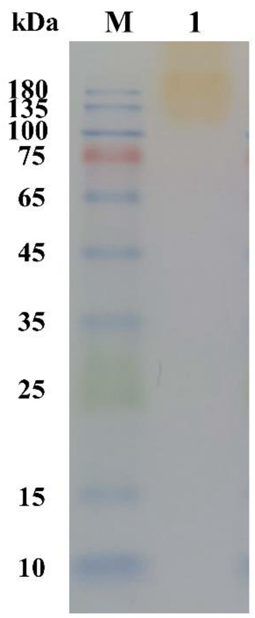

Figure S2. The native PAGE analysis of *St*MCO from *S. thermocarboxydus*. Lane M, the protein molecular mass marker; lane 1, the purified recombinant *St*MCO.

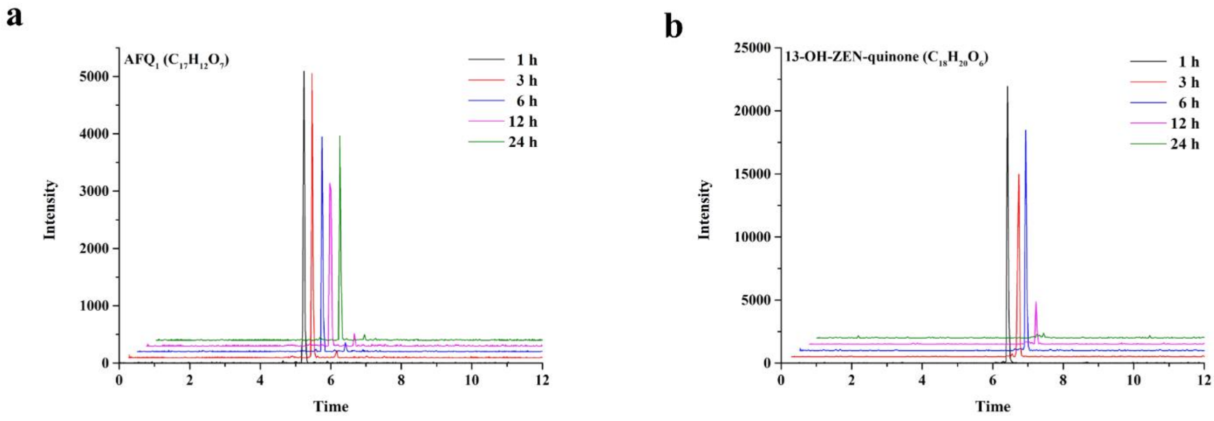

Figure S3. The time-course analysis of AFB_1 and ZEN degradation products by UPLC-MS/MS, including AFQ_1 (**a**) and 13-OH-ZEN-quinone (**b**).
